# Exploring UAS-lidar as a sampling tool for satellite-based AGB estimations in the Miombo woodland of Zambia

**DOI:** 10.1186/s13007-024-01212-4

**Published:** 2024-06-08

**Authors:** Hastings Shamaoma, Paxie W. Chirwa, Jules C. Zekeng, Able Ramoelo, Andrew T. Hudak, Ferdinand Handavu, Stephen Syampungani

**Affiliations:** 1 Forest Science Postgraduate Programme, Department of Plant and Soil Sciences, University of Pretoria, Private Bag X20, Hatfield, Pretoria, 0028 South Africa; 2 Department of Urban and Regional Planning, Copperbelt University, 21692 Kitwe, Zambia; 3 Department of Forest Engineering, Advanced Teachers Training School for Technical Education, University of Douala, P.O. Box 1872, Douala, Cameroon; 4 Oliver R Tambo Africa Research Chair Initiative (ORTARChI), Chair of Environment and Development, Department of Environmental and Plant Sciences, Copperbelt University, 21692 Kitwe, Zambia; 5 Centre for Environmental Studies (CFES), Department of Geography, Geoinformatics and Meteorology After CFES, University of Pretoria, Private Bag X20, Hatfield, Pretoria, 0028 South Africa; 6 Forestry Sciences Laboratory, USDA Forest Service, Rocky Mountain Research Station, 1221 South Main St., Moscow, ID 83843 USA; 7 Department of Geography, Environment and Climate Change, Mukuba University, 20382 Kitwe, Zambia

**Keywords:** Above ground biomass, UAS-lidar, Two-phase, Sampling tool

## Abstract

To date, only a limited number of studies have utilized remote sensing imagery to estimate aboveground biomass (AGB) in the Miombo ecoregion using wall-to-wall medium resolution optical satellite imagery (Sentinel-2 and Landsat), localized airborne light detection and ranging (lidar), or localized unmanned aerial systems (UAS) images. On the one hand, the optical satellite imagery is suitable for wall-to-wall coverage, but the AGB estimates based on such imagery lack precision for local or stand-level sustainable forest management and international reporting mechanisms. On the other hand, the AGB estimates based on airborne lidar and UAS imagery have the precision required for sustainable forest management at a local level and international reporting requirements but lack capacity for wall-to-wall coverage. Therefore, the main aim of this study was to investigate the use of UAS-lidar as a sampling tool for satellite-based AGB estimation in the Miombo woodlands of Zambia. In order to bridge the spatial data gap, this study employed a two-phase sampling approach, utilizing Sentinel-2 imagery, partial-coverage UAS-lidar data, and field plot data to estimate AGB in the 8094-hectare Miengwe Forest, Miombo Woodlands, Zambia, where UAS-lidar estimated AGB was used as reference data for estimating AGB using Sentinel-2 image metrics. The findings showed that utilizing UAS-lidar as reference data for predicting AGB using Sentinel-2 image metrics yielded superior results (Adj-R^2^ = 0.70, RMSE = 27.97) than using direct field estimated AGB and Sentinel-2 image metrics (R^2^ = 0.55, RMSE = 38.10). The quality of AGB estimates obtained from this approach, coupled with the ongoing advancement and cost-cutting of UAS-lidar technology as well as the continuous availability of wall-to-wall optical imagery such as Sentinel-2, provides much-needed direction for future forest structural attribute estimation for efficient management of the Miombo woodlands.

## Introduction

Sustainable management and carbon accounting of forests require accurate up-to-date vegetation structural data, often covering extensive areas that are too huge to capture, process, and manage by manual methods [1–6]. Typically, above ground biomass (AGB) in the Miombo woodlands is determined using destructive harvesting procedures, for building allometric equations based on the observed data from these cut trees, such as diameter at breast height (DBH), tree height, and wood density [7–9]. Nevertheless, the application of these allometric equations on extensive forest regions can pose challenges in terms of time, cost, and feasibility due to the difficulty in obtaining field measurement input parameters in remote terrains. Consequently, the AGB for most of vegetation formations in many parts of the African savannas, Miombo woodlands inclusive remains poorly understood.

Remote sensing has made it possible to measure vegetation structure across vast areas in an efficient and repetitive manner [10, 11]. The application of remote sensing methods in estimating AGB in the Miombo woodlands [12–18] is becoming common. Most of these studies employ statistical models where field estimates of AGB are regressed against metrics generated from corresponding remote sensing data, followed by extrapolation of resulting models to the entire study area. The studies that have employed remote sensing imagery for estimation of AGB in the Miombo ecoregion so far have done it at two levels of abstraction, namely: (i) wall-to-wall estimation of AGB; and (ii) local or stand-level estimations. The wall-to-wall category includes, the use of atmospherically resistant vegetation indices (ARVI) and normalized difference vegetation indices (NDVI) derived from Landsat imagery to assess forest cover, stocking and above-ground tree biomass dynamics in the Miombo woodlands of Tanzania [14]. In another study, Halperin et al. [12, 19] estimated AGB in Nyimba district, Miombo woodlands, Zambia, using National Forest Inventory (NFI) data, estimated canopy cover, environmental data, disturbance data, and Landsat 8 OLI satellite imagery. The medium resolution imagery (Landsat) utilized in Kashindye et al. [14] and Halperin et al. [12, 19] are suitable for wall-to-wall coverage, but the AGB estimates based on such imagery lack precision for local or stand-level sustainable forest management, as well as international reporting mechanisms [20] such as reducing emissions from Deforestation and Forest Degradation, plus forest conservation, sustainable management of forests and enhancement of carbon stocks (REDD +) and Monitoring, Reporting and Verification (MRV), which offers monetary rewards to developing countries for forest conservation, and the execution of ecologically sound forest management based on national carbon stocks reported to the United Nations Framework Convention on Climate Change, UNFCCC [2, 21].

At a local level, Mauya et al. [16] employed airborne light detection and ranging (lidar) data to estimated AGB in the Miombo woodlands of Liwale district, Tanzania. Another study by Kachamba et al. [13], utilized unmanned aerial systems (UAS) image-based point clouds to estimate AGB in the Miombo woodlands, Muyobe forest, and Mzimba District in northern Malawi. The AGB data estimates by Mauya et al. [16] and Kachamba et al. [13] have the precision required for sustainable forest management at a local level and international reporting requirements but lack capacity for wall-to-wall coverage. Furthermore, apart from the limited area coverage inherent in the UAS imagery approach employed in Kachamba et al. [13], the imagery requires huge storage space and high processing speeds [22, 23] that are too demanding and still challenging for wall-to-wall estimations of AGB over a large area. As a result, the two levels of abstraction must be linked in order to get wall-to-wall AGB estimates with the accuracy necessary for local sustainable forest management and international carbon reporting requirements [2, 21].

With regard to bridging the spatial gap between wall-to-wall satellite imagery and detailed airborne and UAS imagery, some studies have proposed a two-phase sampling design where areas covered by UAS or airborne imagery are sampled via field plots and areas covered by wall-to-wall satellite images are sampled using UAS or airborne imagery, for example, lidar sampling [24–28] and UAS imagery sampling [29, 30]. These strategies have demonstrated tremendous potential to reduce field plot installation costs and improve wall-to-wall AGB estimate accuracy, which could provide solutions for forest data collection in forest inventory-plagued regions such as the Miombo ecoregion. A study by Wulder et al. [31] presented a complete review of employing lidar sampling to allow large-area forest characterizations, in which lidar samples were utilized in a way comparable to field samples. However, their review focused on airborne, which are still expensive to acquire in the Miombo region. UAS provide a more flexible and affordable sampling platform for use in conjunction with wall-to-wall satellite imagery, as demonstrated in recent studies [28, 30, 32].

In a pioneering study for UAS-based sampling, Puliti et al. [30] used UAS photogrammetric point clouds as a sampling tool, together with a limited sample of field data and wall-to-wall Sentinel-2 images, to estimate growing stock volume in a 7330 hectare forest area in Norway using a hierarchical model-based inference and reported this approach to be cost-effective for large scale forest resource assessments. However, UAS photogrammetric point clouds have been reported to have challenges in capturing the vertical vegetation structure that are required for estimating AGB in denser forest environments [22, 33]. In a related study, Wang et al. [32] used a lidar sensor mounted on a UAS platform (UAS-lidar) partial coverage data as a link between field plot data and wall-to-wall Sentinel-2 imagery to estimate mangrove forests AGB in Hainan Island, China. Apart from lowering field sampling costs, their research observed that their method produced better AGB estimations (R^2^ = 0.62; rRMSE = 35.41%) than the usual method, which directly correlates field plots to Sentinel-2 data (R^2^ = 0.0.52; rRMSE = 39.88%).

This paper proposes a two-phase sampling technique for low-cost, large-scale AGB estimates for the Miombo ecoregion by capitalizing on publicly-available Sentinel-2 satellite images and inexpensive UAS-lidar data. In order to achieve this, the specific objectives were: (i) to identify suitable UAS-lidar metrics and Sentinel-2 metrics for estimating AGB in the Zambian Miombo; (ii) to identify the optimal prediction model for mapping AGB; (iii) to assess if UAS-lidar-estimated AGB can replace field-estimated AGB as reference data; and (iv) to compare the findings of direct field plots to Sentinel-2 AGB estimations from utilizing field plots to UAS-Lidar and UAS-lidar to Sentinel-2 in a two-phase sampling strategy.

## Materials and method

### Study area

**Fig. 1:**
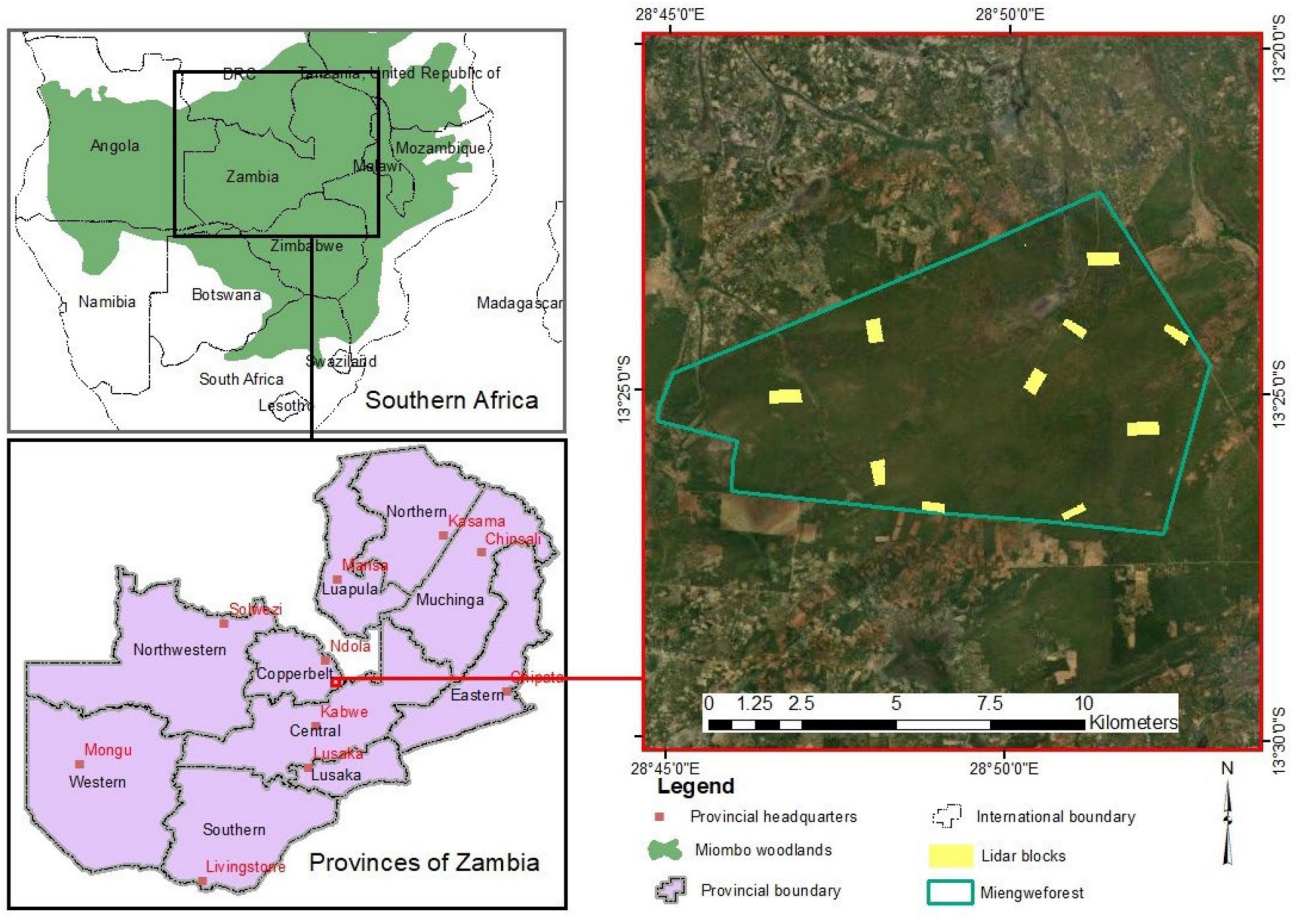
Location of study area

The research was conducted in Miengwe Forest Reserve Number 36, Masaiti District, Copperbelt Province, Zambia (Fig. 1). The forest reserve is situated approximately 17 km from the Ndola-Lusaka highway and 90 km southwest of the Ndola city center. The 8,094-ha Miengwe Forest Reserve is located between 13°24′05′′S and 28°49′00′′E. The region receives an average of 1200 mm of rainfall annually and experiences three distinct seasons: hot dry (September–November), rainy (December–March), and cold dry (April–August) [7]. The most prevalent soil form is residual lateritic soil, which consists primarily of silty clays and sediments. The area is within the Wet Miombo region and is characterized by the dominance of the families of Papilionacae and Fabaceae. The dominant genera and species are Brachystegia (*Brachystegia spiciformis* and *Brachystegia longifolia*), Julbernardia (*Julbernadia globiflora* and *Julbernadia paniculata),* and Isoberlinia (*Isobernilia angolensis*).

### Field sample plots

**Fig. 2:**
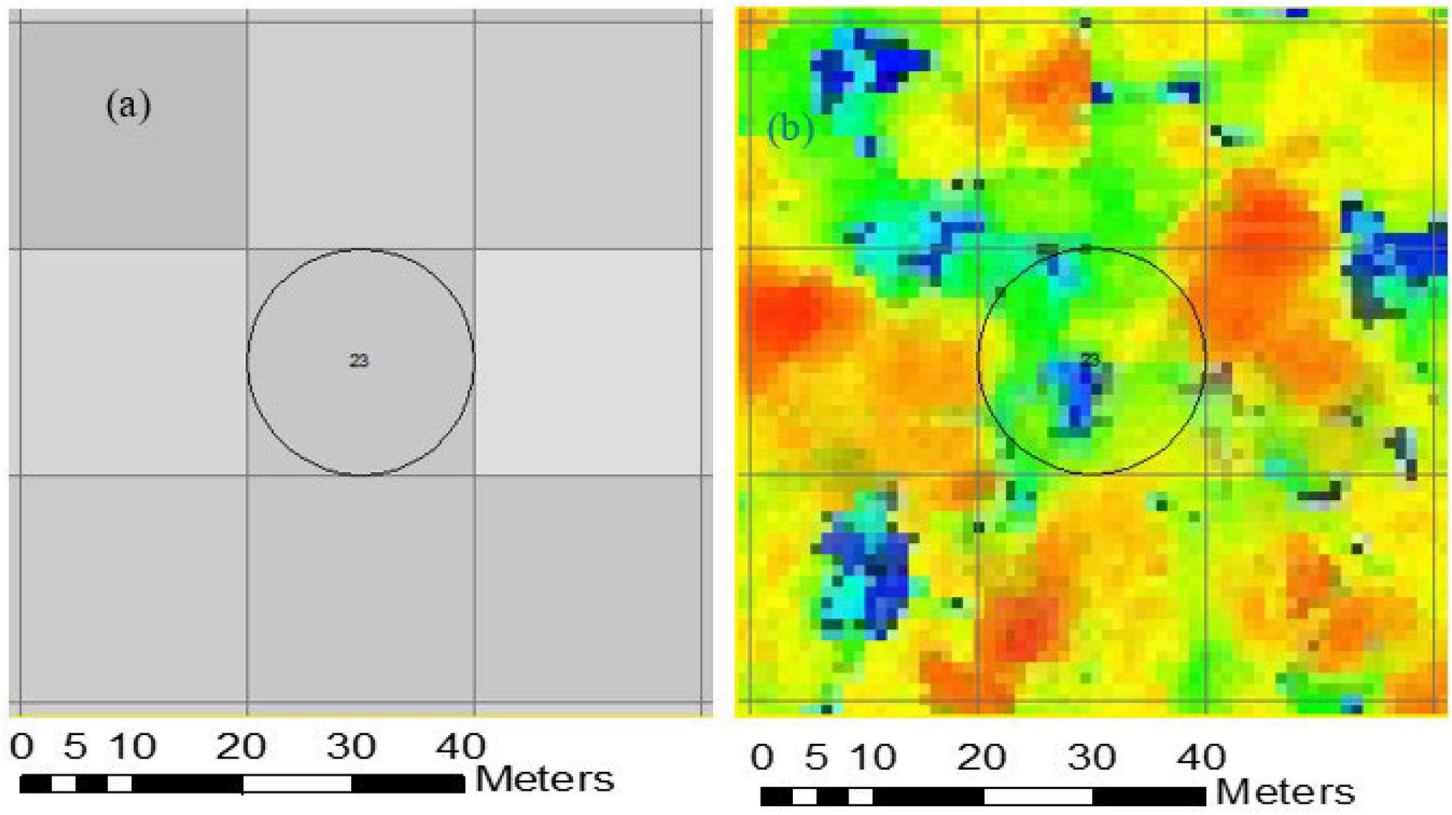
Sample plot and grid framework overlaid on: **a** Sentinel-2 image and **b** lidar point cloud

To ensure that field sample plots, UAS-lidar data, and Sentinel-2 data corresponded in the two-phase sampling approach [34], the Sentinel-2 image covering the study area was resampled to 20 m spatial resolution and used to generate a 20 × 20 m grid framework that served as the foundation for both field and UAS-lidar sampling (Fig. 2). The study area was divided into ten UAS lidar blocks ranging in size from 30 to 50 hectares, which were selected based on the vegetation coverage, accessibility, and availability of a UAS launch site as determined by visual interpretation of Google Earth images and field assessment. In each of the UAS-lidar blocks, ten to twelve circular sample plots of 10 m radius were established at 250 m spacing at the centre of the 20 × 20 m Sentinel-2 grid framework, at least 50 m distant from the block border. These plots were designed to align with the 20 × 20 m grids that were used for extracting UAS-lidar metrics. The LT700H real time kinematic RTK (Shanghai Huace Navigation Technology Limited, China) Global Navigation Satellite Systems (GNSS) receiver was used to precisely locate the centers of these plots on the ground to within a few centimeters. The DBH, tree height, and species names of trees with DBH more than 5 cm were recorded in each of the sample plots. Allometric equations proposed by Handavu et al. [7] were used to estimate AGB at the plot level.

### Collecting and pre-processing data from UAS-lidar

Using a T-Drone M1200 quadcopter equipped with a gAirHawk GS-100C UAS-lidar scanning system, we collected the raw UAV-lidar point clouds between November 10th and 12th, 2021. The Livox Avia sensor on the GS-100C UAS-lidar operates at 200 HZ and can provide up to 720,000 points/sec in triple echo. The mission was planned using the open-source program Mission Planner, which was also used to track the aircraft in real-time and monitor its flight characteristics. UAS-lidar data were collected at an altitude of 80 m, a speed of 5 m per second, and a swath width of 42 m. A GNSS ground base station was used as a reference for subsequent UAS-lidar data post-processing.

The unprocessed UAS-lidar data downloaded from the GS-100C comprised raw lidar points, UAS inertia measurement unit data, UAS GNSS data, and raw photogrammetry imagery (used for colourising the point cloud). The raw UAS-lidar data and raw GNSS data from the ground GNSS base station were first processed in gAirhawk 5.0 version software (Geosun Navigation Technology Limited, Wuhan, China), where lidar data, IMU data, and GNSS base data were integrated to process the flight trajectory and generate georeferenced UAS-lidar point cloud data in las format. The UAS-lidar point cloud data in las format underwent further processing in Lidar360 version 5.4.3.0 software (GreenValley International, California, CA, USA), which included: (i) denoising the lidar point cloud using an outlier reduction method; (ii) classification of point clouds into either ground or non-ground using an enhanced version of the progressive triangulated irregular network (TIN) densification filter method [35]; and (iii) normalizing point clouds by subtracting the elevation of each point from the DTM that was generated using the inverse distance weighting (IDW) interpolation technique. The normalized points were used as input for extracting UAS-lidar metrics which were used for the subsequent modelling.

### Sentinel-2 data collection and pre-processing

There were no cloud-free images for November 2021 to coincide with the lidar data collection period, so Sentinel-2 images with less than 5% cloud cover captured in November 2022, which reflected the closest state to the time lidar data was collected, were downloaded from the open access European Space Agency [36]. The Sentinel Application Platform (SNAP) and ArcGIS Desktop Version 10.7.1 [37] software were used to pre-process the raw Sentinel-2 imagery. The Sen2Cor atmospheric correlation processor (version 2.5.5) was used to do atmospheric correction to create Level2A bottom-of-atmosphere reflectance data. Three visible bands [Blue (B2), Green (B3), and Red (B4)], three red edge bands [Red Edge 1 (B5), Red Edge 2 (B6), and Red Edge 3 (B7)], two near infrared bands (B8) and Narrow Near Infrared (B8a)), and two shortwave bands [Shortwave 2 (B12) and Shortwave 3 (B13)] were used in the Sentinel-2 image composite. Bands 1, 9, and 10 were removed because they were dedicated to atmospheric correction and had coarse resolution of 60 m. All adopted bands were resampled to 20 m resolution using the nearest neighbor approach in ArcGIS to match our sampling strategy [28, 38, 39]. Finally, subsets of all generated sentinel-2 imagery products were clipped to the size of the study area.

### Extraction of AGB predictors

The UAS-lidar metrics were extracted in Lidar360 software based on polygons generated from a 20 × 20 m resampled Sentinel-2 grid framework (Fig. 2). We generated a total of 37 UAS-lidar metrics at the plot level (Table 1). The 20 × 20 m grid framework was generated based on the re-sampled Sentinel-2 using the “create fishnet tool’ in ArcToolbox, implemented in ArcGIS Desktop software, which includes an option for generating points inside each grid. The points inside each grid served as the basis for extracting Sentinel-2 image metrics for estimating the AGB for the study area.

**Table 1:** UAS-lidar metrics

Lidar metrics	Description
Percentile heights (H1, H5, H10, H20, H25, H30, H40, H50, H60, H70, H75, H80, H90, H95, H99)	The percentile of the canopy height distributions (1st, 5th 10th, 20th, 25th, 30th 40th, 50th, 60th, 70th,75th, 80th 90th, 95th and 99th) of first returns
Canopy return density (D1, D2, D3, D4, D5, D6, D7, D8, D9)	The proportion of points above the quantiles (10th,20th, 30th, 40th, 50th and 60th, 70th, 80th and 90th) to total number of points
Variance of height (Hvar)	The variance of the heights of all points
Maximum height (Hmax)	Maximum of return heights above 2 m
Coefficient of variation of heights (Hcv)	Variation of heights of lidar returns above 2 m
Hskew	Skewness of height
Hmd	The median of absolute deviation of heights
Hkurtosis	The kurtosis of the heights of all points
Hstd	Standard deviation of height
Hmean	Mean height above ground of all first returns
Canopy relief ratio (CRR)	Mean height returns minus the minimum height divided by the maximum height minus the minimum height
Canopy cover (CC) above 2 m	Percentile of first returns above 2 m
Gap fraction (GF)	An indication how much of the sky is visible from beneath a plant canopy
Leaf area index (LAI)	Half of the surface area of all leaves per unit ground area

### Acquiring Sentinel-2 metrics

Prior experience [38, 39] in estimating AGB using Sentinel-2 imagery influenced the choice of relevant bands as well as the derived vegetation indices (VI) and biophysical variables (BV) in this work (Table 2). In addition, normalized difference fraction index (NDFI), an index that has been widely used to monitor forest disturbances in the tropics [40–42] was calculated. It is based on spectral unmixing, which is the breakdown of the spectral signature of a mixed pixel into proportions of endmembers (pure spectra) [43]. Using this approach, Souza et al. [41] employed a linear mixture model to decompose field data on cleared, selectively logged, and undisturbed Amazon forests into proportions of soil, shade, green vegetation (GV), and non-photosynthetic vegetation (NPV). Dense forests revealed high GV and low soil, NPV, and shade percentages. Cleared and thinned forests exhibited greater canopy shade and GV than non-disturbed forests. The NDFI was adopted in this study because it emphasizes the difference between forest and non-forest pixels [40], which is crucial for estimating AGB. The NDFI was calculated using Eq. 1 and GV_shade_ is the shade-normalised GV fraction given by Eq. 2 [41].

**Table 2:** Selected multispectral bands, VI, and BF from Sentinel-2 images

Bands	Description	Central wave length (nm)
B2	Blue	490
B3	Green	560
B4	Red	665
B5	Vegetation red edge	705
B6	Vegetation red edge	740
B7	Vegetation red edge	783
B8	Near infrared (NIR)	842
B11	Short wave infrared (SWIR)	1610
B12	Shortwave infrared (SWIR)	2190

Vegetation indices	Description (reference)	Equation
NDVI	Normalized Difference Vegetation Index [76]	$\text{NDVI}=\frac{\text{B}8-\text{B}4}{\text{B}8+\text{B}4}$
EVI	Enhanced vegetation index [76]	$\text{EVI}=2.5\times \frac{(\text{B}8-\text{B}4)}{(\text{B}8+6\times \text{B}4-7.5\times \text{B}2+1)}$
SAVI	Soil adjusted vegetation index [76]	$\text{SAVI}=\frac{\text{B}8-\text{B}4}{\text{B}8+\text{B}4+\text{L}}\times (1+\text{L})$
RENDVI_705	Red-edge normalized difference vegetation index [76]	$\text{RENDVI}=\frac{\text{B}8-\text{B}5}{\text{B}8+\text{B}5}$
NBRI	Normalized Burn Ratio Index [60]	$\text{NBRI}=\frac{\text{B}8-\text{B}12}{\text{B}8+\text{B}12}$
GNDVI	Green Normalized Difference Vegetation Index [77]	$\text{GNDVI}=\frac{\text{B}8-\text{B}3}{\text{B}8+\text{B}3}$

Biophysical variables	Description (reference)	
LAI	Leaf area index [39]	
FAPAR	Fraction of absorbed photosynthetically active radiation [39]	
FCOVER	Fraction of vegetation cover [39]	
CAB	Chlorophyll content in the leaf [39]	

1 $$NDFI=\frac{{GV}_{\text{shade}}-(NPV+Soil)}{{GV}_{\text{shade}}+(NPV+Soil)}$$

2 $${GV}_{\text{shade}}=\frac{GV}{1+Shade}$$

NDFI is the ratio of the GV, NPV, soil, and shade endmember fractions, with the resulting NDFI values ranging from -1 to 1. In the present study, the calculation of the NDFI was implemented within the System for Earth Observation Data Access, Processing, and Analysis for Land Monitoring (SEPAL) cloud application [44]. Subsequently, the final suitable metrics for the study were arrived at after undergoing a variable section process.

### Predicting AGB

The multi-linear regression (MLR) approach was employed to predict AGB in this study because of its simplicity and ability to handle dependencies on or correlations between the predictor variables [26, 45]. A two-phase sampling approach was utilized to estimate the AGB for the Miengwe forest. The first phase involved creating the ground plot to UAS-lidar relationship and estimating AGB in the blocks covered by UAS-lidar. The UAS-lidar blocks were selected based on accessibility and availability of a UAS launch site and did not follow a strict north–south orientation. Since the UAS-lidar blocks did not match the orientation of the Sentinel-2-generated grid framework, the grid cells in the UAS-lidar block’s margins, covering only a fraction of the 400-m square grid, were removed. We estimated the AGB for a total of 4248 grid cells covering all the 10 UAS-lidar blocks in the study area, representing about 2.5% of the total Miengwe forest area. The estimated AGB of the UAS-lidar blocks were used as reference points in the subsequent estimation of AGB in areas covered by Sentinel-2 imagery for the rest of the study area.

In the second phase, a relationship was established between the UAS-lidar predicted AGB (response variable) and wall-to-wall Sentinel-2 image metrics (Table 2) as predictor variables to estimate the AGB for the entire study area using MLR technique. Seven hundred random (700) points were generated within the 10 UAS-lidar blocks using the create random points tool implemented in ArcGIS Desktop Version 10.7.1. The 700 random samples of UAS-lidar estimated AGB grid cells served as training data for estimating AGB for the whole study area using Sentinel-2 image metrics.

We also predicted the AGB for the Miengwe forest using the direct relationship between ground plots and Sentinel-2 imagery metrics, which allowed us to assess whether or not the use of UAS-lidar as a bridging sampling tool between the two was beneficial. The UAS-lidar to Sentinel-2 estimated AGB was later compared with the one obtained directly the ground points to Sentinel-2 metrics estimated AGB.

### The MLR modeling approach

The first stage of variable selection involved using Pearson's correlation coefficient (r) to evaluate the association between the dependent variable and the independent variables to ensure model parsimony and eliminate overfitting by removing predictor variables with high levels of correlation with each other (r > 0.85). The best subsets regression approach built in Minitab Version 21.1.1 [46] was used to identify the best performing model and variables from a set of selected variables. As a model selection method, best subsets regression involves trying out every conceivable collection of predictor variables and picking the one that performs the best statistically [47]. The best model is chosen based on different criteria including: highest adjusted-R^2^ and predicted-R^2^ as well as the lowest values for Mallows Cp, Akaike's Information Criterion corrected (AICc), and Bayesian information criterion (BIC). In our case the model with lowest AICc was considered to be the best as it has been proved to perform well for smaller samples in prior studies [48, 49]. Finally, the best MLR model was used to predict the AGB.

To compare the predicted values with the observed values (AGB values acquired from lidar), three accuracy assessment indicators employed in Liu et al. [50] were utilized. The developed MLR models were tested using k-fold cross validation to determine their accuracy. The idea behind this method is to randomly divide the data into k groups or folds where each member is nearly the same size. When doing k-fold cross-validation, each fold is treated as its own validation set. We choose k = 10 because this number has been widely used and empirically proved to provide non-biased and rather stable estimates of the test error rate. Ten subsets of the original dataset are created and used for tenfold cross-validation. Each fold uses 9 of the 10 subsets for training and the remaining 1 for testing the accuracy of the learnt model on the validation set. Each subgroup will undergo the validation procedure many times. Finally, we utilized the aforementioned equations to calculate cross-validated RMSE from a table containing all of the folds' predicted values.

## Results

### Variables selection

In this study, three models were developed to predict AGB in two phases: Model 1, represented by Eq. 3, utilized the correlation between field estimated AGB and UAS-lidar metrics. Model 2, represented by Eq. 4, utilized the correlation between UAS-lidar estimated AGB and Sentinel-2 metrics. Model 3, represented by Eq. 5, was developed by utilizing the direct correlation between field estimated AGB and Sentinel-2 image metrics for the purpose of comparing with model 2. Since the processes for models 1–3 are similar, we only show the variable selection process for model 1. The variable CC emerged as the primary predictor in all ten models identified in the best subsets approach, indicating its significant influence (Table 3). Hcv and H80 were also shown to be influential predictors, since they were picked in seven out of the ten models. Overall, height related metrics dominated the list of selected lidar metrics.

**Table 3:** Candidate MLR Models for Field estimated AGB prediction using UAS-lidar metrics (see Table 1 for UAS-lidar metrics description)

Vars	R^2^	adj-R^2^	pred-R^2^	Cp	RMSE	AICc	BIC	CC	Haad	H20	H30	H80	H99	Hcv	D10	D20	D30	D70
1	0.66	0.65	0.56	37.7	0.19441	28.629	27.312	X										
2	0.79	0.77	0.53	20	0.16265	21.062	20.189	X		X								
3	0.84	0.82	0.74	3.9	0.12312	8.524	7.814	X		X	X							
**4**	**0.89**	**0.87**	**0.82**	**1.6**	**0.10209**	**0.995**	**0.122**	**X**	**X**	**X**	**X**							
5	0.90	0.87	0.82	0	0.10318	4.08	2.664	X	X	X	X						X	
6	0.90	0.87	0.79	1.5	0.10399	7.401	4.995	X	X	X	X				X	X		
7	0.90	0.87	0.77	3.2	0.10564	11.59	7.663	X	X	X	X			X	X	X		
8	0.90	0.86	0.76	5.1	0.10835	16.841	10.755	X	X	X	X			X	X	X	X	
9	0.90	0.85	0.67	7	0.11127	22.837	13.819	X	X	X	X	X	X	X		X	X	
10	0.90	0.84	0.54	9	0.11481	29.93	17.027	X	X	X	X	X	X	X		X	X	X

X: selected variable, bold values: selected model

The model of four predictor variables was chosen to be the best model because it produced the highest predicted R^2^ and lowest AICc (Bolded in Table 3), and was less complicated compared to the model of six predictor variables. After implementation of the chosen model, it resulted in model 1, Eq. 3. This selection procedure was repeated in phase two for estimating UAS-lidar-derived AGB using Sentinel-2 metrics and resulted in model 2, Eq. 4 (Table 4). The same procedure was applied to directly estimate AGB using the relationship between field-estimated AGB and Sentinel-2 metrics, resulting in model 3, Eq. 5

**Table 4:** Candidate MLR Models for UAS-lidar estimated AGB prediction using Sentinel-2 metrics (see Table 2 for Sentinel-2 metrics description)

Vars	R^2^	adj-R^2^	pred-R^2^	Cp	RMSE	AICc	BIC	NDFI	B02	NBRI	B06	B11	B07	B05	B12	B04	LAI
1	46.3	46.3	45.2	268.2	0.66021	1493.583	1507.379			X							
2	56.1	56.0	54.8	87.0	0.59750	1346.480	1364.864	X		X							
3	58.8	58.7	57.3	38.1	0.57911	1301.127	1324.092	X		X	X						
4	59.7	59.5	58.1	23.2	0.57311	1286.692	1314.234	X		X	X				X		
5	65.3	65.1	62.8	13.8	0.56914	1277.416	1309.529	X		X			X		X		X
**6**	**78.7**	**70.4**	**63.9**	**9.5**	**0.56615**	**1273.136**	**1309.914**	**X**		**X**	**X**		**X**		**X**		**X**
7	78.8	70.4	63.9	9.4	0.56671	1273.144	1314.382	X		X	X	X	X		X		X
8	78.9	70.5	63.8	9.4	0.56633	1273.184	1318.977	X	X	X	X		X	X	X		X
9	79.0	70.5	63.7	9.0	0.56579	1272.816	1323.157	X	X	X	X	X	X	X	X		X
10	79.0	70.5	63.3	11.0	0.56617	1274.856	1329.741	X	X	X	X	X	X	X	X	X	X

X: selected variable, bold values: selected model

3 $$ln\left(AGB\right)= 1.68CC + 0.08H80 + 5.32D20- 2.97Hcv + 0.20$$

4 $$ln\left(AGB\right)= 4.18NDFI+0.24LAI+5.98NBRI-14.53B06-8.49B12+3.15B11+2.05$$

5 $$AGB=2778B11+1084GNDVI+59.1LAI-1171$$

### AGB estimation at phase one

**Fig. 3:**
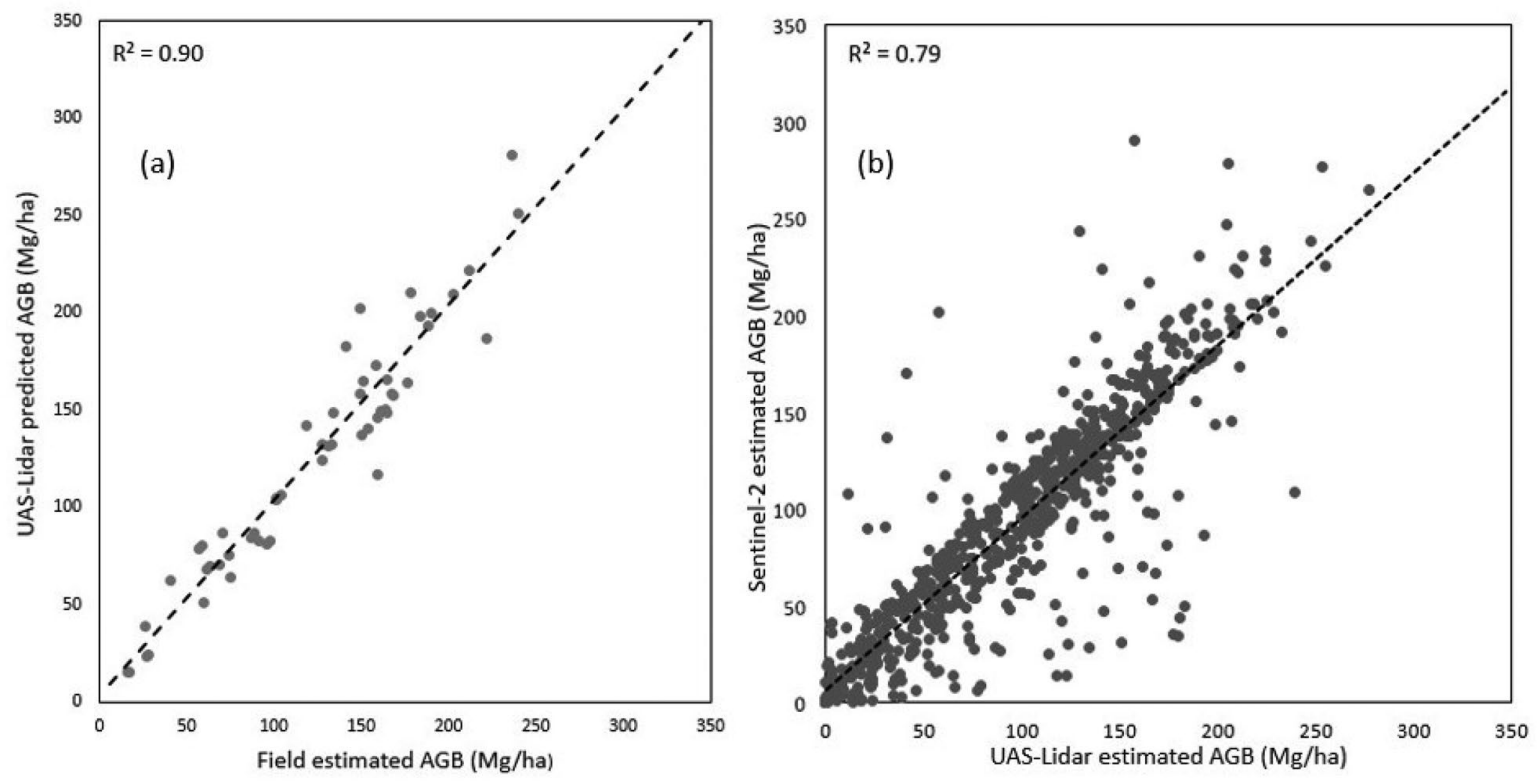
Scatter plots showing estimation of above ground biomass: **a** Ground to UAS-lidar model and **b** UAS-lidar to Sentinel-2 model

Estimation of AGB by applying the relationship between field estimated AGB and UAS-lidar metrics using model 1 explained 90% of the variance of AGB, RMSE of 17.70 Mg/ha and a bias of 3.79 Mg/ha (Table 5) and Fig. 3a, indicating that the model successfully predicted the AGB.

**Table 5:** Summaries of used models

Model	R^2^	adj-R^2^	Pred-R^2^	RMSE (Mg/ha)	rRMSE%	Bias (Mg/ha)
Ground—UAS-lidar	0.90	0.87	0.81	17.70	14.38	3.79
UAS-lidar—Sentinel-2	0.79	0.70	0.64	27.97	28.89	3.94
Ground—Sentinel-2	0.62	0.55	0.46	38.10	37.54	6.19

### AGB estimation at phase two

**Fig. 4:**
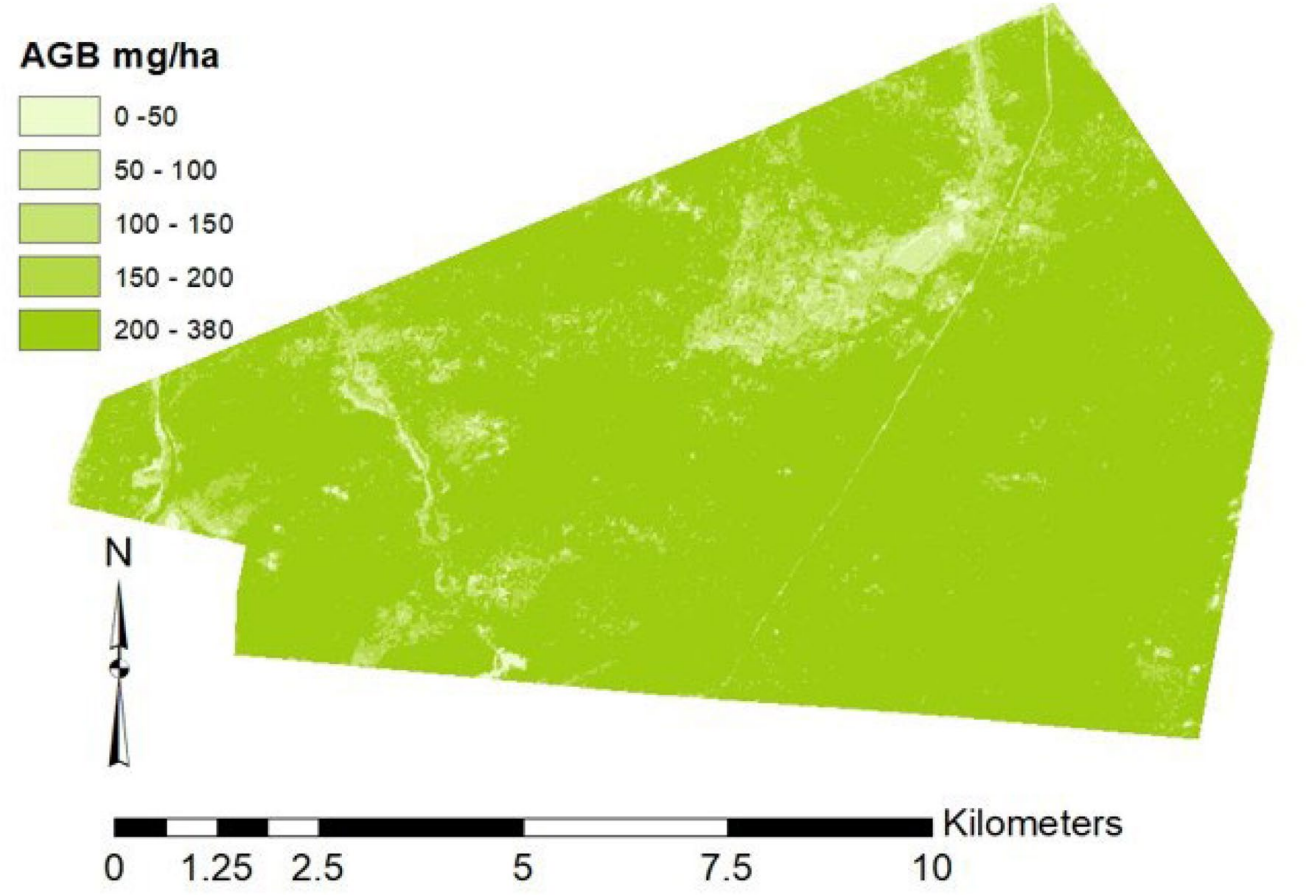
Biomass map for Miengwe forest at 20 m resolution

**Fig. 5:**
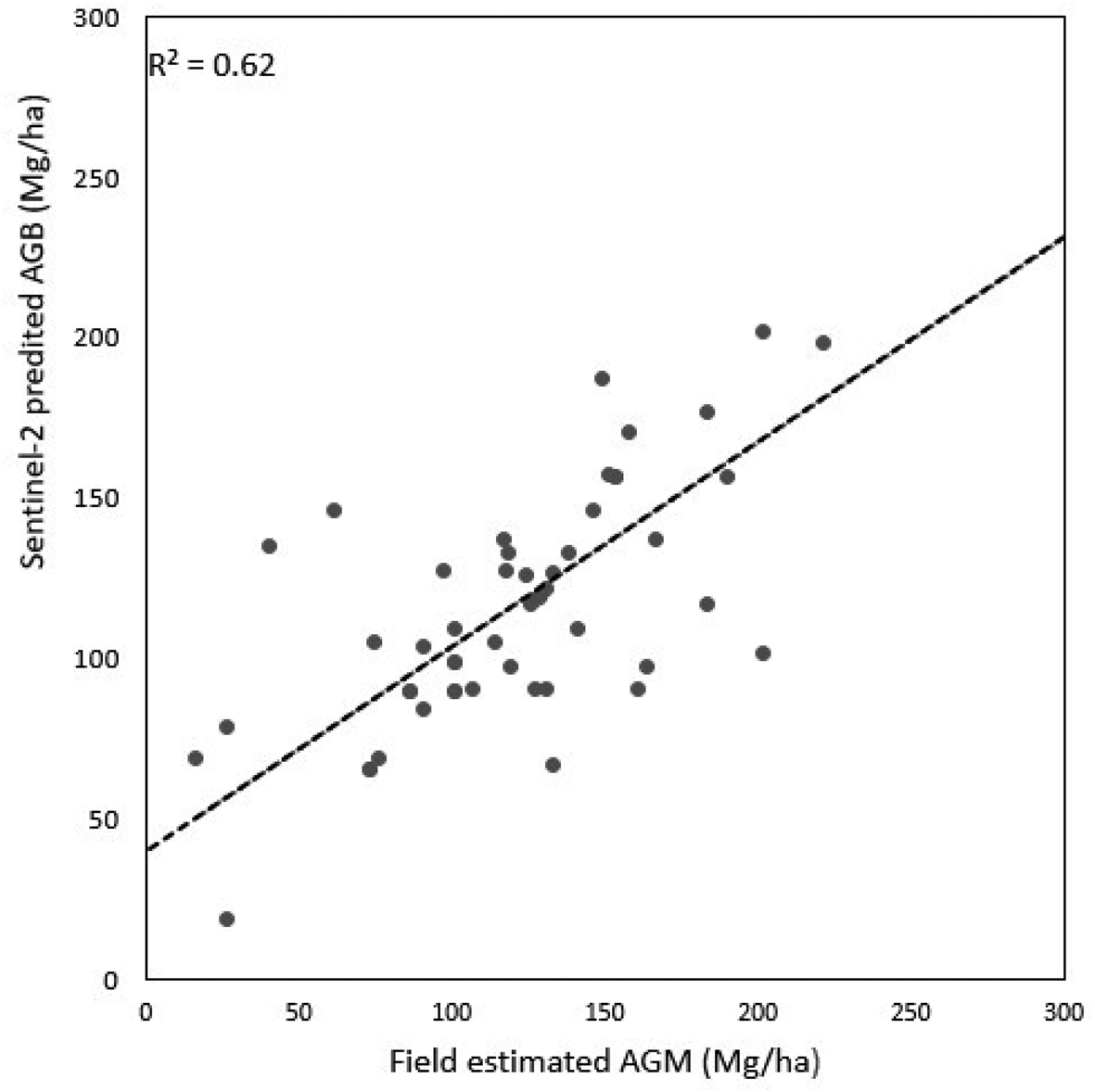
Scatter plots showing estimation of above ground biomass using ground to Sentinel-2 model

In phase 2, UAS-lidar prediction of AGB from phase one were used as sample data for predicting AGB using the relationship with Sentinel-2 variables (Eq. 4, model 2) and was able to explain 79% of the variance of AGB for the entire Miengwe forest. Additionally, model 2 was used to generate the AGM map at 20 m resolution for the Miengwe forest (Fig. 4). The RMSE of 27.97 Mg/ha and bias of 3.94 Mg/ha was achieved (Table 4) and Fig. 3b. With a cross-validated predicted R^2^ = 0.64, this demonstrated potential for applying UAS-lidar sampling when estimating AGB using Sentinel-2 imagery, contrasting it with what was determined using usual direct ground sampling to Sentinel-2 metrics, explaining only 62% of the variance of AGB across the Miengwe forest and a cross-validated predicted R^2^ = 0.46 Table 5 and Fig. 5). The UAS-lidar-Sentinel-2 model exhibited a bias of 3.94 Mg/ha, which was only slightly higher than the bias of 3.79 Mg/ha in the Ground-UAS-lidar model, showing a good match between Sentinel-2 and UAS-lidar data and validating the use of UAS-lidar sampling.

## Discussion

Accurately estimating AGB across extensive forest areas presents a significant challenge. Currently, AGB estimates for the majority of the vegetation formations of the Miombo woodland remain unknown, and corresponding AGB maps for these areas are unavailable. The present study presents the approach for producing an AGB map (Fig. 3) for Miombo woodland through utilization of a two-phase UAS-lidar sampling methodology that leverages the combined advantages of field plots, UAS-lidar technology, and Sentinel-2 imagery.

### Choosing the optimal predictors for estimating the AGB

The process of variable selection was conducted in order to identify the optimal predictors for accurately estimating the AGB across all phases. The first phase involved selecting best predictors for estimating AGB using the relationship between field estimated AGB and UAS-lidar derived metrics. The second phase involved selecting best predictors for estimating AGB using the relationship between UAS-lidar metrics estimated AGB and metrics derived from Sentinel-2 imagery. The third and final phase was to select the best Sentinel-2 metrics for predicting AGB using the relationship between AGB estimated through field observations and Sentinel-2 image metrics.

In phase 1, the most important predicators for AGB were a set of metrics associated with height, density, and canopy cover. CC was the most important predictor selected in all the 10 models for predicting AGB, followed by Hcv and H80, which were selected in 7 of the 10 models (Table 3). This accords with UAS-lidar metrics selected in previous studies elsewhere, for example, height percentiles [50–52], canopy cover [50, 51], canopy density [51] and coefficient of variation for heights [50, 53, 54] for estimating AGB. Several previous studies [50, 55, 56] have demonstrated the utility of Hmean as a predictor for estimating aboveground biomass (AGB). However, in our study, it was seen that Hmean exhibited a strong correlation with other predictors, and as a result, it was excluded from further consideration. The differences in the selected predictors can be attributed to variation in metric selection algorithms, modelling approach and variation in forest structure and composition [57–59].

For phase 2, the best Sentinel-2 image metrics predictors for estimating AGB were vegetation indices (NDFI and NBRI), the red-edge band (B6), SWIR bands (B11 and B12) and the biophysical variable LAI. The vegetation indices (NDFI and NBRI) and red-edge bands (B7 and B6) were strong for models with fewer predictor variables (models 1–3, Table 4) because they are known to be good for separating vegetated from non-vegetated areas [39–41, 60], which is critical for AGB estimation. The red edge band lies at a specific wavelength that fluctuates swiftly at the convergence of the near-infrared and red spectral bands [57]. This band is highly responsive to subtle changes in both the structure of the plant canopy and the chlorophyll content. Consequently, it is regarded as having the capacity to mitigate saturation effects and improve estimation of AGB, supporting works by other researchers (e.g. [61]). Furthermore, this supports an assertion by Adam et al. [62] that vegetation indices possess the ability to mitigate the effects of shadows and environmental factors on reflectance, thereby enhancing their correlation with AGB. The addition of the SWIR bands and the biophysical variable LAI resulted in improved models (models 5–10, Table 4). This finding is consistent with previous studies conducted by Dang et al. [63] in Yok Don National Park, Vietnam, Mauya and Madundo [38] in tropical montane forests of Tanzania, and Moradi et al. [64] in Zagros oak forests in Iran, who reported a high correlation between AGB and red, red-edge, NIR and SWIR bands and vegetation indices that are derived from them.

B11, NDFI, and LAI were the selected predictors (Eq. 5) for directly calculating AGB using field-estimated AGB and Sentinel-2 measurements. This was consistent with the results of Muhe and Argaw [39], who employed Sentinel-2 metrics to estimate AGB in a tropical afro-montane forest in Ethiopia. However, unlike Muhe and Argaw [39], Sentinel-2-derived biophysical variables were observed to be significantly correlated with each other, and just LAI was utilized to develop the model as opposed to the three biophysical variables applied in Muhe and Argaw [39]. Sentinel-2 derived products (indices and biophysical factors) were added instead of raw Sentinel-2 bands only since they were shown to enhance AGB estimates in previous research [39, 65]. The NDFI was a strong predictor in both models 2 and 3. This is not surprising because this index has been observed to be good at discriminating vegetated from non-vegetated areas [40, 41]. In addition to selecting a suitable regression model, the variable selection strategy approach was crucial to lowering the feature dimension, minimizing information redundancy, and enhancing modeling efficiency [47].

### Identify the optimal prediction model for mapping AGB

After choosing the most important predictors for estimating AGB at the two phases, best subsets regression [47], was used to come up with the best models for predicting AGB at all phases (Tables 3 and 4). Our criteria were based on the model with the highest prediction accuracy (pred-R^2^) as well as the lowest AICc, BIC and Mallows Cp, followed by the model with the fewest predictors, in that order. However, the most important consideration in selecting the optimal model was checking to see whether it contains variables that are consistent with ecological reasoning and have been shown to be strong AGB predictors in the literature [66]. The model included height metrics including the lower, middle, and upper percentiles, thereby offering data on the distribution of tree heights, as well as metrics for canopy cover and density, thus yielding valuable insights into canopy cover. Previous studies have shown the efficacy of using the complement of selected metrics in estimating AGB [50, 52, 53]. Our approach aligns with prior research that utilized the best subsets regression method, which was determined to be efficacious in identifying the optimal multiple linear regression (MLR) model [47, 52].

### Model comparison

Model 1 (Eq. 3), in which we estimated the AGB using the relationship between field AGB estimates and UAS-lidar metrics, yielded the best results overall (Adj-R^2^ = 0.84, rRMSE = 14.7%). It outperformed models 2 and 3, which predicted AGB using Sentinel-2 metrics. This is not surprising considering that lidar data, unlike optical images represents 3-dimensional vegetation structure [67, 68]. Model 1 also performed better than Mauya et al. [16], who estimated AGB using airborne-lidar in the Miombo woodlands of Tanzania (rRMSE 46.8%). Point cloud densities may have caused the variation in AGB estimate accuracy [16]. The airborne-lidar system utilized in Mauya et al. [16] had an average point density of 1.8 pts m^−2^, whereas the UAS-lidar employed in this study had 300 pts m^−2^. Since canopy height determination relies on the DTM, a greater point density will result in a better terrain surface model and more accurate canopy height determination [69–71]. Model 2 (Eq. 4) used the relationship between UAS-lidar estimated AGB from model 1 with Sentinel-2 image metrics to estimate the AGB for the entire study area, achieving (Adj-R^2^ = 0.7, rRMSE = 28.9%), which was obviously less precise than model 1, but achieved better results than model 3, which used direct relationship between field estimated AGB and Sentinel-2 metrics to estimate AGB. These findings confirms work by Wang et al. [32], who employed UAS-lidar and Sentinel-2 imagery to estimate AGB in mangrove forests, northeastern Hainan island, China. The better performance of model 2 can be attributed to the large number of UAS-lidar estimated AGB reference points as well as the sampling strategy (Fig. 2), which precisely linked the UAS-lidar data and Sentinel-2 data to a common location on the ground.

### UAS-lidar as reference data

Previous research has shown that utilizing UAS imagery data to replace field data as reference data in a two-phase sampling approach is feasible [30, 32]. This was demonstrated in this study when UAS-lidar estimated AGB was used as reference data to estimate AGB using Sentinel-2 imagery for the entire study area, achieving (Adj-R^2^ = 0.70), comparable to a study by Mauya et al. [16] who used airborne-lidar to estimate AGB in the Miombo woodlands of Tanzania and achieved (Adj-R^2^ = 0.69). The positive relationship between UAS-lidar estimated AGB and Sentinel-2 image metrics exhibited in this study has benefits with synergistic potential to improve AGB estimation in the Miombo ecoregion. On the one hand, UAS-lidar offers the benefits of flexible deployment, affordability, and the capacity to capture precise vertical structure of vegetation, but it has drawbacks in terms of poor area coverage and massive processing and storage memory requirements [22, 72]. On the other hand, we have multi-spectral Sentinel-2 imagery, which is suitable for wall-to-wall coverage at 10 m resolution with NIR, red-edge, and SWIR bands, and a short revisit period of 5 days that it is useful for AGB estimation but falls short of capturing the fine vertical vegetation structure details that are required for forest management at a local level [2, 21]. The findings of this study validate UAS data’s capacity to deliver comprehensive training and validation information, which would have otherwise taken a significant amount of time and money utilizing field inventory processes. Furthermore, Sentinel-2-based AGB estimation offers a viable technique for broadening the scope of assessments beyond UAS-surveyed areas, boosting the efficiency of AGB estimation and monitoring operations. Previous research conducted on the estimation of AGB in the Miombo forests using direct ground to medium resolution Landsat data has shown suboptimal model fit. Kashindye et al. [14] found R^2^ values ranging from 0.47 to 0.65 in their research conducted in Babati district, Tanzania. Their finding falls within a similar range as the study conducted by Halperin et al. [19] in Nyimba district, Zambia, where the R^2^ ranged from 0.35 to 0.59 and it agrees with what was found utilizing direct ground to Sentinel-2 estimation in the present study (R^2^ = 0.62). These were all lower than the estimations derived in this work by ground-UAS-Lidar-Sentinel-2 two-phase sampling (R^2^ = 0.79). Hence, the integration of the two remote sensing data sources, as exemplified in this research, in conjunction with field techniques, enables the estimation of AGB in the Miombo woodlands with comprehensive accuracy that surpasses the individual capabilities of either data source, as evinced in prior studies [32, 73, 74].

### Benefits of two phase-sampling

Estimation of AGB across vast Miombo woodlands is often restricted by the difficulty in obtaining sufficient field measurements owing to a variety of reasons such as limited labour, limited financial resources, remoteness, and poor access to their location. Most Miombo woodlands AGB estimation studies are undertaken over small regions or at a local scale using either destructive sampling [7, 8, 75] or remote sensing methods [13, 16] and a modest number of field samples. The two-phase sampling approach has demonstrated how UAS-lidar could be used to upscale the field sampling to cover extensive areas, even with few field sample plots. From a modest 54 field points in phase 1, we were able to upscale to 700 UAS-lidar sample points in phase 2 to cover extensive areas and easily relate between UAS-lidar estimated AGB and Sentinel-2 metrics to estimate AGB over an expanded area covered by the Sentinel-2 image. The benefits of using the upscaling UAS-Lidar-Sentinel-2 imagery model (adj-R^2^ = 0.70) as opposed to the direct field Plots-Sentinel-2 imagery model (adj-R^2^ = 0.55) to estimate AGB have been demonstrated. The reason for an improved result from the UAS-lidar sampling technique could be because UAS-lidar covers a larger area with more points representing a wide range of vertical and horizontal vegetation structural changes and accurately measures terrain morphology. Then, using the UAS-lidar estimated AGB as training samples, the model can fit AGB variations over the entire study area and generate high prediction accuracy. This assertion is supported by earlier studies that employed lidar as a sampling tool for biomass estimation [25, 26, 32]. Though not investigated in this study, earlier studies have demonstrated that UAS-lidar sampling reduces the required number of field samples and the overall sampling cost [30, 32]. Previous research, however, has shown that optical Sentinel-2 images may become saturated in densely forested regions. This saturation problem may negatively impact AGB estimations. Nonetheless, Wang et al. [78] showed that adding Sentinel-1 synthetic aperture radar (SAR) data might assist reduce saturation and improve AGB estimates over wide regions. They did so by using data from UAS-Lidar, Sentinel-1, and Sentinel-2 satellites to estimate AGB for regional coniferous forests in China. Similarly, Navarro et al. [29] estimated AGB in Senegalese mangrove plantations using UAS-SfM point clouds, Sentinel-1, and Sentinel-2 data. The outcomes of this research and other related studies suggest that this technique can be used for improved AGB estimation for the entire Miombo ecoregion.

Arguably, the best approach could have been using most accurate UAS-lidar to estimate the AGB for estimating the AGB for the whole study area. But UAS-lidar has limitations in terms of area covered per flight, storage space and processing speed [22, 23], which makes it cumbersome to cover extensive areas. In the present study, for example, the coverage area achieved during each flight utilizing our UAS was limited to 30–40 hectares. Moreover, the point clouds from flight (one UAS-lidar block) required 30–40 Giga Bites (GB) of storage space for processing. These factors provided a substantial challenge for our field laptop, which had just 150 GB of free space, restricting us to processing three blocks at a time and backing them up to an external drive before moving on to the next batch. With all of the aforementioned problems and what the literature has adequately stated [22, 23], it can be concluded that the utilization of UAS-lidar technology is currently limited to small sites and can only serve as a sampling tool for larger sites.

## Conclusion

A two-phase sampling approach was used to estimate total AGB in the Miengwe forest reserve in the Miombo woodlands of Zambia. The findings of this study show the potential of using UAS-lidar as a sampling tool for estimating and monitoring AGB and other forest structural attributes across vast regions using wall-to-wall Sentinel-2 imagery when field data are limited. The AGB estimates are of a precision that is suitable for local forest management and international reporting mechanisms such as REDD + and MRV. The approach used in this study could be up-scaled to provide spatially consistent, low cost and precise AGB estimates over extensive regions for supporting the long-term sustainability of carbon monitoring and reporting initiatives in Miombo woodlands. The continuous improvement and reduction in cost of UAS-lidar technology and the continuous availability of wall-to-wall optical imagery such as Sentinel-2 assure viability and warrant further investigation and refinement of this approach for future wall-to-wall carbon monitoring and reporting programs in the Miombo ecoregion.

## Data Availability

The data that support the findings of this study are available from the corresponding author upon reasonable request.
